# Beyond Pneumonia: Autoimmune Pulmonary Alveolar Proteinosis in a Pediatric Patient

**DOI:** 10.7759/cureus.107798

**Published:** 2026-04-27

**Authors:** Jessica Satei, Tarek Husien, Ibrahim Abdulhamid

**Affiliations:** 1 Pediatrics, Detroit Medical Center (DMC) Children's Hospital of Michigan, Detroit, USA; 2 Pediatric Pulmonology, Central Michigan University College of Medicine, Detroit, USA

**Keywords:** abnormal stat5, anti-granulocyte-macrophage colony-stimulating factor antibodies, autoimmune pulmonary alveolar proteinosis (pap), bal lavage, pediatrics lung fibrosis

## Abstract

Pulmonary alveolar proteinosis (PAP) is a rare pediatric lung disorder marked by the accumulation of surfactant-like material within the alveoli, impairing normal gas exchange. Among its subtypes, the autoimmune form, caused by neutralizing antibodies against granulocyte-macrophage colony-stimulating factor (GM-CSF), is particularly uncommon in children and often mimics atypical infections. We describe the case of a 12-year-old girl with progressive dyspnea, hypoxia, and declining physical performance over four months. Imaging revealed diffuse bilateral ground-glass opacities, and initial treatment for atypical pneumonia showed minimal improvement. Further workup, including bronchoalveolar lavage and lung biopsy, identified alveolar spaces filled with periodic acid-Schiff (PAS)-positive proteinaceous material. Serologic testing confirmed elevated GM-CSF autoantibodies, establishing the diagnosis of autoimmune PAP. The patient required supplemental oxygen and underwent multiple whole lung lavages (WLL), ultimately achieving significant clinical improvement and functional recovery. This case underscores the importance of considering autoimmune PAP in pediatric patients with unexplained interstitial lung disease unresponsive to conventional therapy. Early testing for GM-CSF autoantibodies and timely initiation of WLL may prevent misdiagnosis and lead to favorable outcomes.

## Introduction

Pulmonary alveolar proteinosis (PAP) is a rare form of lung disease characterized by the accumulation of surfactant-derived lipoproteinaceous material within the alveoli, resulting in impaired gas exchange and progressive respiratory dysfunction [1,2]. Autoimmune PAP variant is the most prevalent, accounting for over 90% of cases in adults [3,4]. The autoimmune form is driven by the presence of granulocyte-macrophage colony-stimulating factor (GM-CSF) autoantibodies, which impair alveolar macrophage maturation and surfactant clearance [5].

Although PAP is well documented in adults, its occurrence in children is exceedingly rare, particularly the autoimmune form. The clinical presentation is often nonspecific, including exertional dyspnea, hypoxemia, and diffuse radiographic abnormalities, frequently mimicking pneumonia or interstitial lung disease [6]. Infectious agents such as *Mycoplasma pneumoniae, Nocardia, Pneumocystis jirovecii, and *human immunodeficiency virus (HIV) have been reported in association with PAP, raising questions about their role as possible immunologic triggers or opportunistic infections in immunocompromised lungs [7,8].

Here, we present a rare case of autoimmune PAP in a previously healthy 12-year-old girl with a confirmed *M. pneumoniae* infection. This case highlights the diagnostic challenges of pediatric PAP, explores a possible infectious contribution to disease onset, and further emphasizes the clinical course, including progression and subsequent remission over five years following the initial presentation.

## Case presentation

A 12-year-old girl presented to the emergency room with difficulty breathing and hypoxia, with an oxygen saturation of 89% on room air. On further questioning, the patient reported exercise intolerance, fatigability, and declining school performance over the past four months. She was previously healthy, with normal growth; her weight and height were at the 76th and 97th percentiles, respectively. She denied any allergies or use of medications, including bronchodilators. Family history was negative for chronic lung diseases.

**Figure 1:**
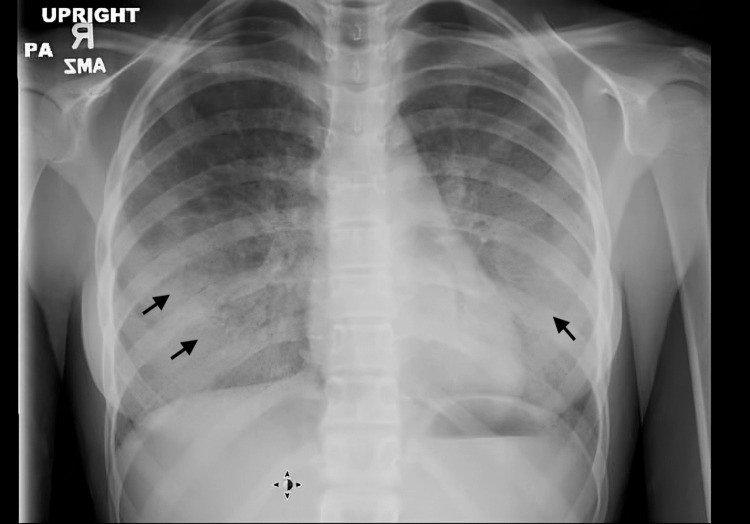
Chest radiograph showing bibasilar infiltrates and interstitial markings

**Figure 2:**
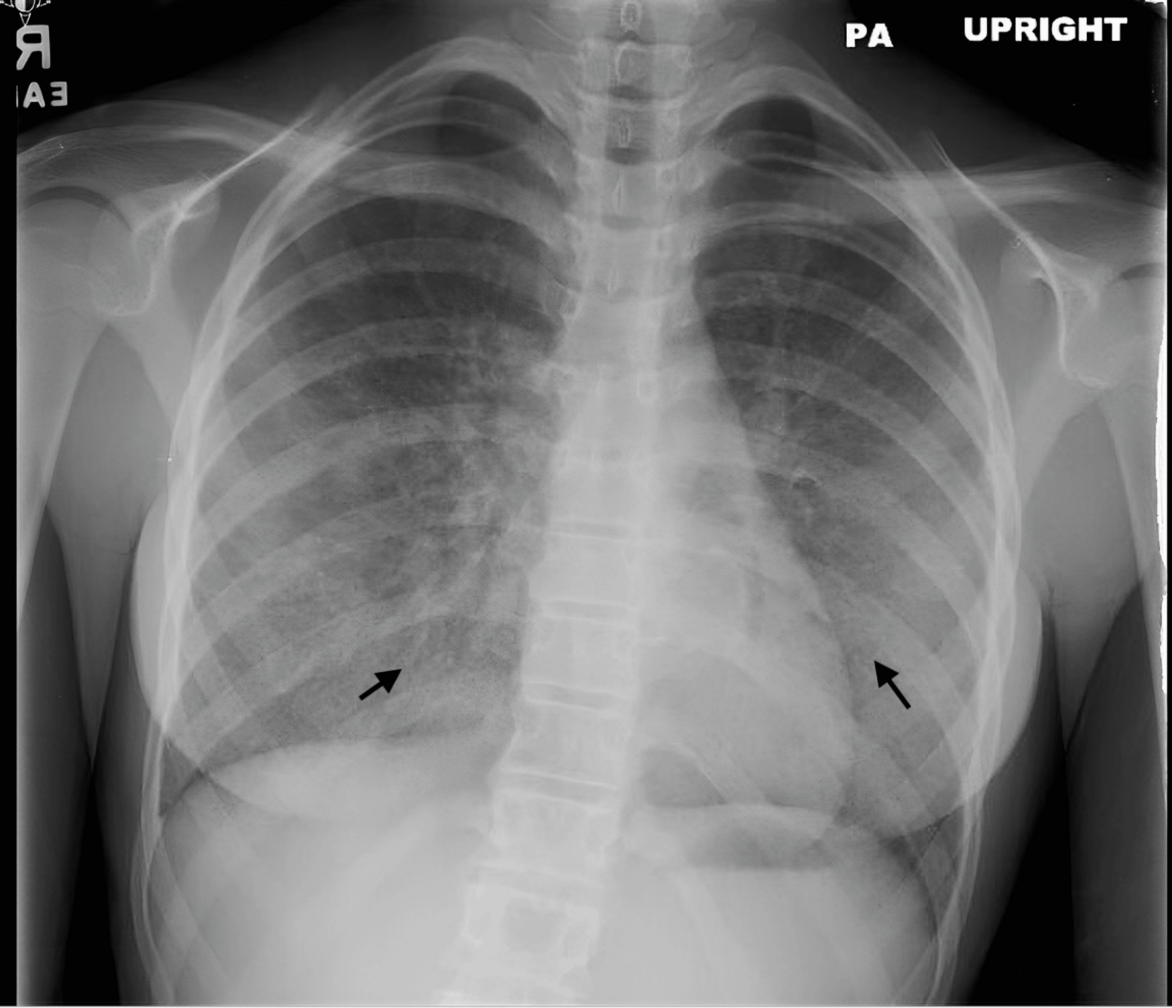
Resolved basilar infiltrates with some persistent interstitial prominence

The patient had been seen in the emergency room one month earlier for a similar complaint of dry cough and shortness of breath. At that time, chest radiography showed bibasilar infiltrates and interstitial markings (Figure 1). A clinical diagnosis of atypical pneumonia was made, and she was treated with azithromycin, a five-day course of oral corticosteroids, and aerosolized albuterol. A follow-up chest X-ray showed resolution of the basilar infiltrates, with some persistent interstitial prominence (Figure 2).

Physical examination revealed tachypnea at rest, with a few bilateral basal inspiratory crackles. Continuous pulse oximetry showed intermittent hypoxemia, and oxygen therapy was initiated.

**Figure 3:**
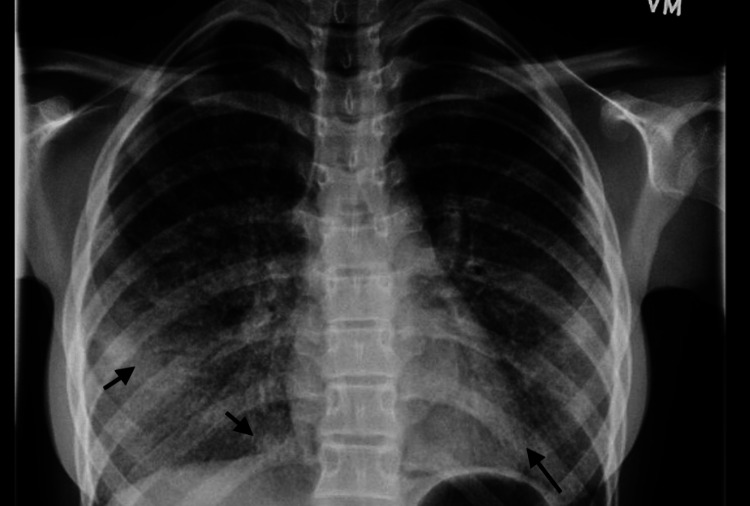
Diffuse reticulonodular opacities in the lower lobes, suggestive of a nonspecific interstitial process, with possible chronic fibrosis and interstitial lung disease

**Figure 4:**
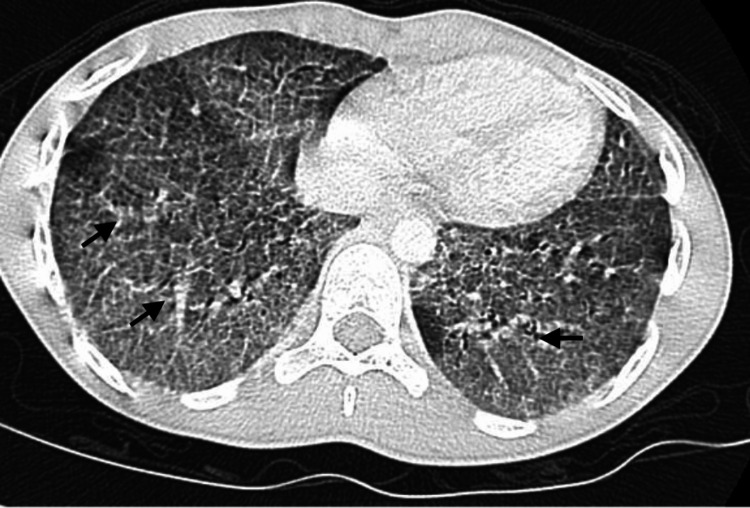
Diffuse ground-glass opacity seen predominantly in the lower lobes, with interlobular septal thickening and bilateral hilar adenopathy

Laboratory workup showed a complete blood count with mild leukocytosis and neutrophil predominance (white blood cell (WBC) 12,000/µL; absolute neutrophil count (ANC) 2,000/µL). C-reactive protein was mildly elevated at 25 mg/L. Respiratory syncytial virus (RSV) and influenza enzyme immunoassay (EIA) tests were negative. A chest X-ray demonstrated diffuse reticulonodular opacities in the lower lobes, suggestive of a nonspecific interstitial process; chronic fibrosis and interstitial lung disease (ILD) were considered (Figure 3). A contrast-enhanced chest CT showed diffuse ground-glass opacities predominantly in the lower lobes, along with interlobular septal thickening and bilateral hilar adenopathy (Figure 4).

Due to the chest CT findings, Pediatric Pulmonology and Infectious Disease were consulted. An infectious workup was largely negative, except for weakly positive *Mycoplasma* IgM titers (1:80), and the patient was treated with a five-day course of azithromycin. However, she remained oxygen-dependent and continued to report dyspnea on exertion. A bronchoalveolar lavage (BAL) and lung tissue biopsy were performed. These showed generalized marked distention of the alveoli and bronchioles with eosinophilic granular material that was PAS-positive and diastase-resistant. Occasional cholesterol clefts were observed within the alveolar contents. Only focal collections of foamy macrophages were present in a few alveoli. The alveolar septa appeared unremarkable, with only hypertrophy of type II pneumocytes noted. These findings were characteristic of alveolar proteinosis. There was no evidence of pulmonary fibrosis or pneumonic infiltrate. No organisms suggestive of *P. jirovecii* were identified on Gomori methenamine silver (GMS) staining. Testing for surfactant protein C and ABCA3 variants was negative, making inherited surfactant dysfunction disorders unlikely.

As suspicion for an autoimmune process increased, a serum sample was sent to an out-of-state laboratory for further evaluation. Results showed an elevated serum GM-CSF autoantibody concentration of 60.4 mcg/mL (reference range: <5.0 mcg/mL). The STAT5 phosphorylation index test was also abnormal, suggesting impaired GM-CSF signaling in leukocytes. These findings were consistent with autoimmune pulmonary alveolar proteinosis (PAP).

Once the diagnosis was established, the patient was discharged home on supplemental oxygen, requiring 1-2 L via nasal cannula to maintain oxygen saturation above 90%. Pulmonary function tests obtained after discharge, while on home oxygen, showed an FEV₁ of 57% predicted. To manage chronic respiratory symptoms, she was started on daily inhaled corticosteroids and as-needed albuterol for bronchodilation. A sleep study, performed due to concerns about nocturnal desaturation, revealed frequent episodes of hypoxia during sleep, supporting the need for continuous oxygen therapy and close respiratory monitoring.

Following diagnosis, the patient experienced multiple hospital admissions due to recurrent pulmonary complications, including dyspnea, pneumonia, and wheezing. To manage worsening hypoxia, she underwent three separate whole-lung lavages with normal saline over a two-year period, which resulted in symptom improvement and reduced oxygen dependence. Despite the chronic nature of her condition, longitudinal follow-up showed gradual clinical improvement. After several years, she demonstrated signs of remission, with stable pulmonary function, including an FEV₁ of 82%. She also reported no dyspnea on exertion, indicating meaningful recovery of functional respiratory capacity.

Although the patient was subsequently lost to follow-up, her most recent visit with pediatric pulmonology four years later showed no evidence of residual disease, dyspnea, or recurrent hospitalizations. She reported only intermittent use of an albuterol metered-dose inhaler.

## Discussion

PAP is an uncommon pulmonary condition caused by the accumulation of surfactant within the alveoli, which impairs oxygen diffusion. This disorder was first described in the medical literature in 1958 [1].

PAP can be broadly classified into autoimmune and non-autoimmune forms. The autoimmune form, which accounts for approximately 90% of cases, is characterized by the presence of autoantibodies against GM-CSF. These antibodies impair GM-CSF signaling, resulting in dysfunctional and immature alveolar macrophages that are unable to effectively clear surfactant from the alveolar spaces. Clinically, this form is typically seen in older children and adults and often follows an indolent course, presenting with progressive dyspnea, cough, and characteristic radiographic findings.

The non-autoimmune category (approximately 10%) includes both congenital and secondary forms. Congenital PAP, which usually presents in infancy or early childhood, results from genetic mutations affecting surfactant proteins or components of the GM-CSF receptor pathway, with either autosomal dominant or recessive inheritance. These cases are often more severe, frequently leading to early respiratory compromise and requiring intensive supportive care, with limited response to standard therapies such as whole lung lavage (WLL). In contrast, secondary PAP develops due to underlying conditions such as hematologic malignancies, infections, or environmental exposures that impair macrophage function [2,4]. Compared with congenital diseases, secondary forms have a more variable clinical course and may improve with treatment of the underlying condition.

In our case, dyspnea and exercise intolerance were the main presenting symptoms, prompting further diagnostic evaluation. Persistent interstitial markings on chest X-ray several months after empiric treatment for atypical pneumonia raised concern for chronic interstitial lung disease versus a rheumatologic process.

Our patient tested positive for *M. pneumoniae* on respiratory PCR. Studies suggest that *Mycoplasma* infection is often observed in PAP; however, it remains unclear whether it acts as a trigger, a complication, or an incidental finding. Infections such as cytomegalovirus, *Mycobacterium tuberculosis*, *Nocardia *species, and *P. jirovecii* have also been reported in association with PAP. In several cases, abnormalities in the number or function of alveolar macrophages were noted, suggesting a potential contributory role in disease pathogenesis [5-7].

It has been proposed that *M. pneumoniae* may bind directly to surfactant protein A (SP-A), facilitating persistent adhesion to type II alveolar cells, macrophages, and histiocytes [8]. This interaction may disrupt normal macrophage function and trigger an immune response, leading to the production of GM-CSF autoantibodies, thereby contributing to the development of autoimmune PAP.

Our patient underwent WLL one year later. Lavage was performed with 1 L of saline in each lung until the effluent became clear. The lavage fluid was noted to be turbid and cloudy, and cultures were negative. The patient tolerated the procedure well. To date, the outcome has been satisfactory; she remains on 1.5 L of supplemental oxygen at night and room air during the day, and she attends school regularly. The patient will continue to require multidisciplinary care. WLL has demonstrated clinical effectiveness in managing PAP, particularly in adolescents and adults, with reported improvement rates ranging from 60% to 84% [9,10]. In selected cases, rituximab has been used as an alternative therapy, with benefit reported in a limited number of adult case studies [11].

Autoimmune PAP generally carries a favorable prognosis when managed with WLL, with five-year survival rates reported to be as high as 95% [12]. Earlier case series suggested that spontaneous resolution might occur in up to 50% of patients [13,14]; however, more recent and larger cohort studies indicate that this is much less common, likely occurring in fewer than 10% of cases. Despite overall positive outcomes, mortality still occurs in a subset of patients, most commonly due to complications such as hematologic disorders (33%), infections (25%), respiratory failure (25%), and hemorrhagic events (13%) [12].

## Conclusions

Autoimmune PAP remains a rare and under-recognized cause of interstitial lung disease in children, often masquerading as more common infectious or inflammatory conditions. This case underscores the importance of maintaining a high index of suspicion when faced with persistent hypoxia and characteristic imaging findings unresponsive to standard therapies. By highlighting this case, we aim to raise awareness of autoimmune PAP among clinicians, especially since early diagnosis and intervention can significantly alter the disease trajectory. While some patients respond well to WLL and supportive care, others may experience a more protracted course requiring repeated interventions and close monitoring. The clinical spectrum and prognosis of pediatric autoimmune PAP can vary widely, and timely recognition is critical to improving long-term outcomes. Greater familiarity with this condition may help reduce delays in diagnosis and prevent unnecessary or ineffective treatments.
